# Endoscopic papillectomy of major papilla lesions: Single tertiary care center experience

**DOI:** 10.1055/a-2684-0042

**Published:** 2025-08-25

**Authors:** Bertrand Napoleon

**Affiliations:** 1 Endoscopy Unit, Hopital Prive Jean Mermoz Ramsay Sante, Lyon, France



This retrospective single-center trial 1 included 51 patients treated by endoscopic papillectomy (EP): 11 familial adenomatous polyposis (FAP), 35 sporadic adenoma/adenocarcinoma lesions (SAL), and five non-adenomatous lesions. The authors aimed to evaluate predictors for recurrence and adverse events (AEs). In addition, they compared baseline characteristics and outcomes between patients with FAP vs SAL. The conclusions of this trial must be compared with results of large trials and guidelines recently published.

As observed in other trials, 10% of the resected specimens were finally neither adenoma nor adenocarcinoma. This is consistent with the difficulty for the pathologist in differentiating adenoma with low-grade dysplasia (LGD) and regenerative tissue. To overcome this issue, the European Society of Gastrointestinal Endoscopy 2 recommends histological confirmation by endoscopic biopsies in case of LGD adenoma before initiating any treatment (strong recommendation, low quality of evidence).

The comparison between patients with FAP syndrome and those with sporadic lesions must be interpretated with caution, given the small number of patients in this trial (11 FAP). In a recent multicenter European trial 3 including a large cohort of patients, propensity-score matching identified 101 patients with FAP and 101 with SAL. Some results were comparable to this series. Patients in the FAP group were mainly asymptomatic (79.2% vs. 46.5%, *P* < 0.001), AEs were not statistically different between the two groups, with pancreatitis and bleeding being the most common, and the recurrence rate was higher in the FAP group (3-year disease-free survival was 76.8% vs 84.8%) but occurred later (median of 25 vs. 2 months in SAL patients). On the other hand, the initial R0 resection rate differed between the two groups (63.4% in the FAP group vs 83.2% in the SAL group) and was higher than in this trial (45% in the two groups) 3 . The low R0 resection rate in this study does not support the conclusion of Suryawanshi et al to consider it as a predictive factor of the absence of recurrence.

Some points must also be highlighted in management of ampullary adenoma in FAP syndrome. An ampullary adenoma smaller than 1 cm with only LGD on biopsy can be resected or followed. The prognosis for the upper digestive tract depends on ampulla lesions but also on gastric and duodenal lesions. The decision to undertake endoscopic treatment must take into consideration the other duodenal (Spigelman classification) or gastric lesions. Upper digestive tract follow-up must be continued even in case of absence of ampullary adenoma recurrence after 5 years. Due to the rarity and specificity of FAP and other rare adenomatous polyposis syndromes, patients should be managed at specialized centers.

The AE rate was comparable to other trials (delayed bleeding and acute pancreatitis 13.7% each). The authors found a correlation between delayed bleeding and pancreatitis, which needs to be confirmed in a large trial. Actually, it is not consistent with a large trial that included 307 patients 4 . In that trial, delayed bleeding represented the most common AE and occurred in 44 patients (14.3%). Multivariate analysis identified oral anticoagulant agents (odds ratio [OR] 4.37, range 2.86–5.95) and procedural bleeding (OR 2.22; range 1.10–4.40) as independently related to delayed bleeding. In patients without procedural bleeding, oral anticoagulant agents (OR 5.63, range 2.25–9.83) and ampullary tumor size (OR 1.07, range 1.01–1.13) were independently related to delayed bleeding. Interestingly, delayed bleeding occurred after a median of 1 day and in 88% of cases within the first 48 hours after EP 4 . A 48-hour hospital stay is consequently systematically planned in many centers to manage the majority of AEs during hospitalization.

In their conclusion 1 , Suryawanshi et al considered EP as safe and effective in removing ampullary lesions. This must be toned down. EP remains a difficult procedure with a higher rate of AEs and a lower rate of R0 resection compared with endoscopic resection of other digestive tumors. Moreover, surgery remains an alternative. In a recent and large matched cohort analysis comparing transduodenal surgical ampullectomy and EP 5 , EP was found non-inferior to the surgical counterpart regarding overall survival, but recurrences and retreatments appeared to be more frequent. Regardless of the chosen treatment, these patients should be managed by experts in specialized centers.
